# Structured mating: Patterns and implications

**DOI:** 10.1371/journal.pgen.1006655

**Published:** 2017-04-06

**Authors:** Ronnie Sebro, Gina M. Peloso, Josée Dupuis, Neil J. Risch

**Affiliations:** 1 Department of Radiology, University of Pennsylvania, Philadelphia, PA, United States of America; 2 Department of Biostatistics, Boston University School of Public Health, Boston, MA, United States of America; 3 National Heart, Lung, and Blood Institute's Framingham Heart Study, Framingham, MA, United States of America; 4 Institute for Human Genetics, University of California, San Francisco, San Francisco, CA, United States of America; 5 Department of Epidemiology and Biostatistics, University of California, San Francisco, CA, United States of America; 6 Division of Research, Kaiser Permanente, Oakland, CA, United States of America; Case Western Reserve University, UNITED STATES

## Abstract

Genetic similarity of spouses can reflect factors influencing mate choice, such as physical/behavioral characteristics, and patterns of social endogamy. Spouse correlations for both genetic ancestry and measured traits may impact genotype distributions (Hardy Weinberg and linkage equilibrium), and therefore genetic association studies. Here we evaluate white spouse-pairs from the Framingham Heart Study (FHS) original and offspring cohorts (N = 124 and 755, respectively) to explore spousal genetic similarity and its consequences. Two principal components (PCs) of the genome-wide association (GWA) data were identified, with the first (PC1) delineating clines of Northern/Western to Southern European ancestry and the second (PC2) delineating clines of Ashkenazi Jewish ancestry. In the original (older) cohort, there was a striking positive correlation between the spouses in PC1 (r = 0.73, P = 3x10^-22^) and also for PC2 (r = 0.80, P = 7x10^-29^). In the offspring cohort, the spouse correlations were lower but still highly significant for PC1 (r = 0.38, P = 7x10^-28^) and for PC2 (r = 0.45, P = 2x10^-39^). We observed significant Hardy-Weinberg disequilibrium for single nucleotide polymorphisms (SNPs) loading heavily on PC1 and PC2 across 3 generations, and also significant linkage disequilibrium between unlinked SNPs; both decreased with time, consistent with reduced ancestral endogamy over generations and congruent with theoretical calculations. Ignoring ancestry, estimates of spouse kinship have a mean significantly greater than 0, and more so in the earlier generations. Adjusting kinship estimates for genetic ancestry through the use of PCs led to a mean spouse kinship not different from 0, demonstrating that spouse genetic similarity could be fully attributed to ancestral assortative mating. These findings also have significance for studies of heritability that are based on distantly related individuals (kinship less than 0.05), as we also demonstrate the poor correlation of kinship estimates in that range when ancestry is or is not taken into account.

## Author summary

We analyzed three generations of whites from the Framingham Heart Study (FHS) using genome-wide genotype data to characterize their genetic ancestry. By examination of spouse-pairs, we observed that individuals of Northern/Western European, Southern European and Ashkenazi ancestry preferentially chose spouses of the same ancestry, however, the degree of endogamy decreased in each successive generation, especially between Northern/Western and Southern Europeans. We then showed that the mating pattern results in Hardy-Weinberg disequilibrium (HWD) at ancestrally-informative SNPs, and also results in linkage disequilibrium (LD) between unlinked loci. The HWD and LD decrease as theoretically expected with the decrease in endogamy noted in each generation. In the FHS sample, spouse genetic similarity can be explained by ancestry-related assortative mating.

## Introduction

The mating pattern determines the genetic structure of a population [1, 2, 3]. The genetic structure of a population refers to the distribution of alleles and genotypes in the population. Characterizing the genetic structure of a study population is important because ignoring it can lead to undetected biases, including false positive findings in genetic association studies [4, 5, 6] and inaccurate estimation of kinship and heritability [7, 8].

Population substructure can arise in a study population from geographic stratification and/or non-random mating. Geographic stratification occurs when the study participants are recruited from different geographic areas, and the genotype data is aggregated for analysis. In this setting, although there may be random mating within each geographic subgroup, there is non-random mating within the aggregated study population. Population substructure can also occur when the study participants are recruited from the same geographic area but individuals are more likely to choose mates with similar genotypes to themselves–which can be a reflection of trait-based or ancestry-related assortative mating [9, 10].

Assortative mating occurs when spouse choice is influenced by some observable phenotype or social characteristic [11]. Phenotype-based assortative mating has been well documented in humans for several traits including age [12, 13, 14], height [12, 15, 16, 17], weight [12, 16] and other physical characteristics such as skin pigmentation [18], and eye and hair color [12, 14]. In addition, there are other behavioral and social factors that are highly correlated between spouse-pairs and are thought to affect mate selection such as educational level [15, 16, 19], occupation [15], socioeconomic status [12,13], religion [12,16], smoking [20], alcohol consumption [21, 22], language [23] and culture [23]. Phenotypic assortative mating generally does not change allele frequencies in the genes related to the trait for which the assortment occurs [24]. However, phenotypic assortative mating leads to both within-locus correlation and between-locus correlation specifically for alleles in the genes underlying the trait or traits that are the basis for the assortment [11] (but not at other alleles). The within-locus correlation changes the distribution of the genotype frequencies for the alleles involved in the assortment towards an excess of homozygosity and Hardy-Weinberg disequilibrium (HWD). The between-locus correlation leads to linkage disequilibrium (LD) between alleles at different loci involved in the assortment, even ones on different chromosomes [2, 24].

By contrast, when assortative mating is related to ancestry (ancestry-related assortative mating), the same phenomena are seen, but in this case, at all loci that differ in allele frequency between ancestral populations, whether they have a phenotypic impact or not [9, 10, 25]. After a single generation of random mating, the within-locus correlation disappears and Hardy-Weinberg Equilibrium (HWE) is achieved [24]. However, the between-locus correlation decays at a slower rate [9]. The correlation (LD) between unlinked loci is halved after a single generation of random mating; more generally, in the case of persistent ancestry-related assortative mating, the rate of decay in each subsequent generation is proportional to ½(1+ ρ), where ρ is the correlation in ancestry between spouse-pairs [9].

While observed Hardy-Weinberg deviations and linkage disequilibrium can be due to a number of factors (e.g. genotyping error, inbreeding), there is a very specific pattern of Hardy-Weinberg deviation and linkage disequilibrium caused by assortative mating. As opposed to inbreeding, which affects all loci equally and creates no linkage disequilibrium, and genotyping error, which presumably is based on technical aspects of genotyping and also has no population pattern, assortative mating only influences the univariate and joint genotype distributions of the loci that are related to the trait for which assortment occurs, and not others. And, there is also a direct relationship between the strength of the association of particular SNPs with the trait undergoing assortment and the degree of HWD and LD expected for them. This relationship was first described for a general, observable trait [24]. We previously extended those formulas to apply to the situation of assortative mating related to genetic ancestry [9] in populations of Latino race/ethnicity, and now apply them here to a three-generation European ancestry population from Framingham, Massachusetts.

The Framingham Heart Study (FHS) is an epidemiologic longitudinal study of several cohorts of participants who reside in Framingham, Massachusetts [26]. Three generations of participants, i.e. the original cohort, their offspring and the offspring’s spouses (offspring cohort), and the original cohort’s grandchildren (third generation cohort) have been studied [26–28]. Some participants of each FHS cohort have been genotyped, first on the Affymetrix 100K single nucleotide polymorphism (SNP) array and more recently on the Affymetrix 500K SNP array [29]. The FHS participants reside in the same geographic area (Framingham, Massachusetts). Therefore any non-random mating seen in the FHS likely reflects true mate preference and social endogamy, which may also be related to local neighborhood demography. This multigenerational study provides us with the unique opportunity to investigate generational changes in the mating pattern.

Here, we analyze all three cohorts of the FHS, to investigate evidence of phenotypic assortative mating between spouses for the physical traits of height, weight, body mass index (BMI) and blood pressure. We also assess the FHS original and offspring cohorts for evidence of ancestry-related assortative mating, evaluate the key genetic factors affecting mate selection and assess how the change in mating patterns over time has affected HWE and LD in subsequent generations.

For comparison, we also examine the genetic consequences of assortative mating for height, a highly heritable trait. However, height is correlated with European genetic ancestry. We investigate the degree to which the genotypic consequences of spouse assortment for height is primarily due to the effect of the contributing SNPs on height versus their correlation with genetic ancestry in the FHS original, offspring and third generation cohorts.

Estimates of kinship between individuals using genome-scale genotype data has become a topic of recent interest. For example, reliably estimating the proportion of complex trait variation that is explained by genome-wide association study (GWAS) SNPs has been a challenging problem in the genetics community. The discrepancy between the heritability of the trait estimated from family-based studies and the proportion of the trait variation explained by common SNPs is termed the “missing heritability” [30]. Recent methods for estimating genetic variance explained by common SNPs employ “unrelated” individuals–i.e. those whose kinship is typically less than .05 [31, 32] Thus, at the heart of estimating the proportion of trait variation explained by all the common SNPs on a genotyping array is kinship estimation. Spouse kinship is also of interest in assessing the impact of assortative mating patterns. Estimates of kinship are influenced by population stratification, because such estimates using SNP data are influenced by population allele frequencies, which may not apply on an individual level [7, 33]. Thus, we evaluate and compare spouse kinship estimates before and after adjusting for genetic ancestry.

## Methods

### Ethics statement

All participants provided written informed consent for genetic analysis, and the study was approved by the Institutional Review Board of Boston University Medical Center. Third party data were used and therefore no participant consent was required for this study. Original ethical approval is available for review from previously published articles [26, 27,28, 34].

### Participants

The participants are from the Framingham Heart Study (FHS) original, offspring, and third generation cohorts. The original cohort was ascertained from the population of Framingham, Massachusetts [26], and subsequent generations of the offspring and the spouses of the offspring (offspring cohort) as well as their offspring (grandchildren of the original cohort) have also been studied [27, 28]. These participants have undergone numerous serial clinical examinations and completed several medical questionnaires. The FHS cohorts have been genotyped using the Affymetrix 500K SNP array [29], and this data was used for the genetic analyses. The FHS cohorts are primarily white and contain a mix of different ethnic white subpopulations characteristic of the greater Boston metropolitan area, including substantial proportions of Northern/Western Europeans (Irish, English), Southern Europeans (Italians), and individuals of Ashkenazi ancestry [29, 35]. Here spouse-pairs were identified by having at least one offspring in the subsequent generation cohort. If an individual had children with more than one partner then we selected the partner who was genotyped. If there was more than one genotyped partner then we randomly selected a single partner. There are numerous spouse-pairs in the original and offspring cohort, which allows for calculation of spouse-pair ancestry correlations as well as calculation of phenotypic correlations for several traits (age, height, weight, BMI, systolic blood pressure (SBP), diastolic blood pressure (DBP)) in the original and offspring cohorts.

**Fig 1:**
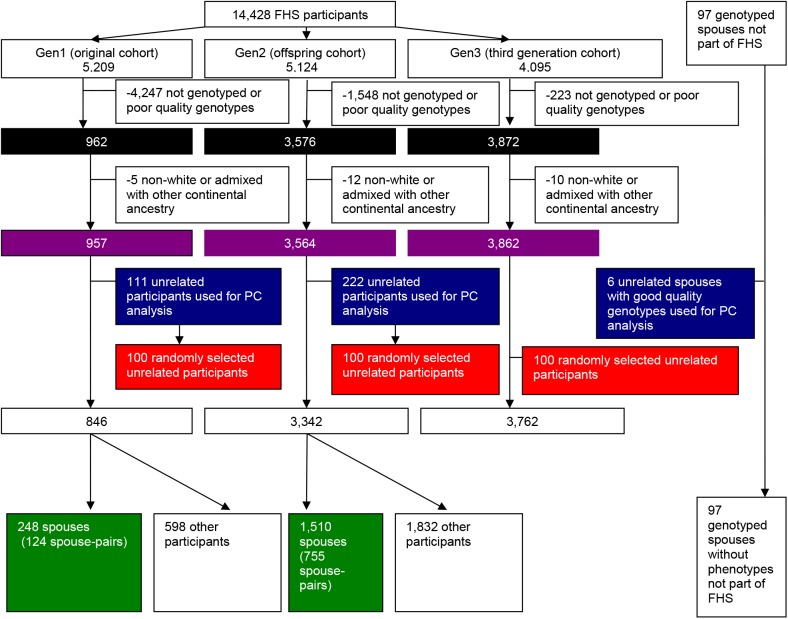
Flow diagram illustrating selection of FHS cohort participants for each analysis. Black box—Participants used to detect global genetic ancestry and identify admixed and non-white individuals. Blue box—Participants used to characterize European/West Asian ancestry. Red box—Participants used for regression analysis using F and D. Green box—Participants used to calculate spousal genetic and phenotypic correlations and used in GCTA and PC-Relate calculations.

This study includes 8,507 participants who had good quality genotypes (less than 3% missing data) from the Affymetrix arrays (described below), with 962 (124 white spouse-pairs) from the original cohort (Gen1); 3,576 (755 white spouse-pairs) from the offspring cohort (Gen2); and 3,872 from the third generation cohort (Gen3), plus an additional 97 spouses of the FHS offspring (Gen2) participants who provided DNA but did not have trait values available and hence did not contribute to the correlation analyses. Fig 1 is a flow diagram illustrating exclusion and selection criteria for the FHS participants for each analysis.

### Genetic markers

**Fig 2:**
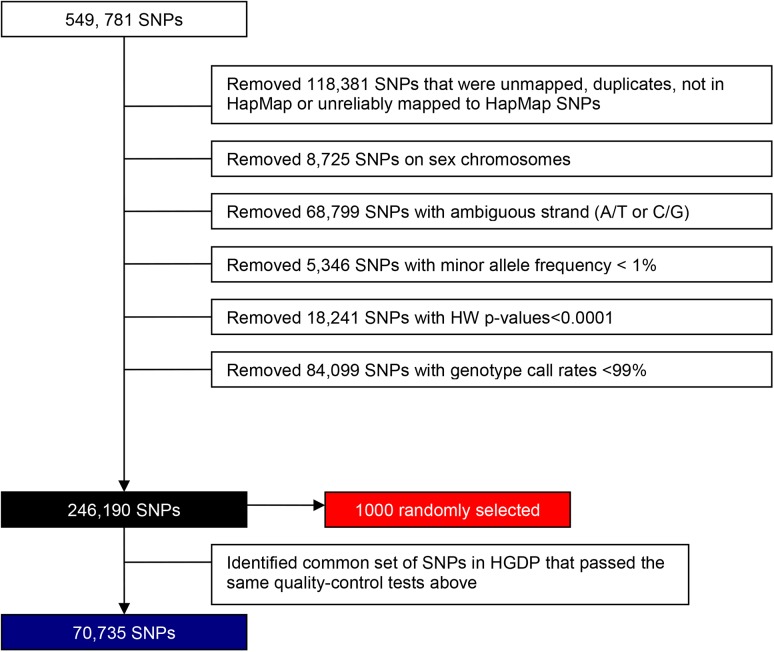
Flow diagram illustrating SNP selection for each analysis. Black box—SNPs used to characterize global genetic ancestry and identify continentally admixed and non-white individuals; SNPs used in GCTA analysis; SNPs used for regression analysis using F; SNPs used to calculate spousal genetic correlations. Blue box—SNPs used to characterize European/West Asian ancestry. Red box—SNPs used for regression analysis using D.

Genotyping was conducted by the FHS SHARe (SNP Health Association Resource) project using the Affymetrix 500K mapping array plus Affymetrix 50K supplemental array. A total of 549,781 SNPs were originally available for analysis, but several quality control (QC) exclusion measures were implemented to insure the highest quality data were used. The SNPs that were excluded were: SNPs that were duplicates, not available in HapMap or not reliably mapped to the HapMap SNPs (118,381); SNPs on the sex chromosomes (8,725); SNPs with ambiguous strand information (A/T or C/G) (68,799); SNPs with minor allele frequency (MAF) < 1% (5,346); SNPs with HW p-values < 0.0001 (18,241); and SNPs with genotype call-rates < 99% (84,099). The 246,190 SNPs that passed these quality-control measures were used for further analyses, including the initial principal components (PC) analysis used to detect global ancestry. Fig 2 is a flow diagram illustrating the SNP selection and exclusion criteria for each analysis.

To interpret the principal components (PCs), we used SNP data from the HapMap [36] and from the Human Genome Diversity Panel (HGDP) [37] for comparison. The HGDP individuals have been genotyped with the Illumina 650Y array [38], whereas the FHS participants had been genotyped with the Affymetrix 500K + 50 K arrays. We identified a common set of 70,735 SNPs that were not duplicates and passed the same quality control measures described above in the FHS, the HapMap data and the HGDP data. These common SNPs were used in the PC analyses to characterize European/West Asian ancestry. The HapMap and HGDP data are available in the National Center for Biotechnology Information (NCBI) Database of Genotypes and Phenotypes (dbGAP).

### Principal components analysis–eliminating non-white study participants

As mentioned above, the majority of the FHS participants are white. Because our analyses focused on the white participants, we first identified participants with evidence of other (e.g. African or Asian) continental genetic ancestry.

We selected the maximum number of unrelated FHS participants for this analysis (521) to ensure the PCs were not confounded or exaggerated by the correlation between relatives. We performed an initial PC analysis of these 521 unrelated FHS participants along with the unrelated individuals from the HapMap (CEPH individuals with Northern and Western European ancestry (CEU), Yoruba from Ibadan, Nigeria (YRI), Han Chinese from Beijing (CHB) and Japanese from Tokyo (JPT)). The PC analysis was performed using the smartpca program from the EIGENSOFT package [39, 40]. The remaining FHS participants were projected onto the first two PCs derived from that analysis. There were no outliers detected when computing PCs with HapMap samples, so outlier exclusion was not performed.

The results revealed that the first PC clearly separated the HapMap participants with European ancestry (CEU) from the participants with African ancestry (YRI), and the second PC clearly separated the participants with Asian ancestry (CHB+JPT) from the participants with European ancestry (CEU). The large majority of FHS participants clustered with the CEU individuals suggesting that these individuals had European ancestry. However, there were a few FHS participants seen to cluster between the CEU and the YRI, consistent with African/European admixture as would be seen in African-Americans. There were also a few participants identified who clustered between the HapMap CHB + JPT and CEU individuals, likely representing admixed participants with European and Asian (or Native American) ancestries. A total of 27 admixed and/or non-white participants were identified and excluded from the subsequent analyses.

### Principal components analysis–characterizing European/West Asian ancestry

A second PC analysis was used to delineate European/Middle Eastern ancestry in the FHS. To help provide geographic orientation to the results, HGDP individuals were projected onto the results of these PC analyses. Because we were investigating the genetic ancestry and phenotypic correlation between spouse-pairs and to eliminate any bias from inclusion of relatives and both members of each spouse-pair in the PC analysis, we selected the maximum number of unrelated individuals (339) including 333 FHS participants (111 from Gen1, 222 from Gen2) and 6 spouses of the FHS participants who provided DNA but were not part of the FHS. When we selected unrelated individuals from the 97 spouses of the FHS participants who provided DNA but were not part of the FHS, only 6 of the spouses were selected. For the other 91 spouses, a relative (child) was already selected for inclusion so these 91 spouses were omitted. Again, the PC analysis was performed using smartpca in the software package EIGENSOFT [39, 40]. There were no outliers identified, so the PC analysis was performed without outlier exclusion. The remaining FHS participants (including members of spouse-pairs) were projected onto the first two PCs from this analysis. These first two PCs were used as measures of the FHS participants’ genetic ancestries. Plots of PC2 versus PC1 were generated for each cohort to assess changes over time/generations.

Price et al. [39] recommended not filtering SNPs to remove SNPs in high LD with each other, so this PC analysis was based on 70,735 markers that were not filtered to remove nearby SNPs with high LD. However, as an additional quality control check, we performed LD pruning and removed 20,129 markers with LD r^2^>.30 so that among the remaining 50,606 markers, no two had an r^2^ greater than .30. The PC scores for the two top PCs derived with the reduced set of markers (50,606) gave virtually identical results to the analysis with the full marker set (70,735) (correlation, r = .998 for PC1 and correlation, r = .978 for PC2).

### Characteristics of the FHS cohorts

FHS spouse-pair demographics were collected at the time of participant recruitment into the FHS (baseline exam) including age, weight, height, BMI, SBP and DBP.

### Spouse correlations

The total number of white spouse-pairs in this analysis was 879, with 124 from the original cohort (Gen1) and 755 from the offspring cohort (Gen2). No information on spouse-pairs was available from the third generation (Gen3) cohort. Spouse correlations for anthropometric measures (height, weight, BMI), age, SBP and DBP were calculated. The eigenvalues/scores from the first and second PCs from the PC analysis were used as measures of genetic ancestry. Quantitative evidence of ancestry-related assortative mating was obtained by examining spouse correlations for PC scores in the original and offspring cohorts. In addition, scatterplots of PC scores for husbands versus wives for PC1 and PC2, in both the original cohort and the offspring cohort were assessed to determine trends in the mating pattern.

### Impact of ancestry-related assortative mating on Hardy-Weinberg equilibrium and linkage disequilibrium

To assess the impact of ancestry-related assortative mating on the genetic structure of the FHS, we calculated the standardized homozygote excess parameter F [9] and the unstandardized linkage disequilibrium (LD) parameter D [41] for pairs of unlinked markers across three generations of FHS. For a locus with alleles A and a, the formula for F is given by:

$$F=(4N_{AA}N_{aa}-N_{Aa}{}^{2})/[(2N_{AA}+N_{Aa})(2N_{aa}+N_{Aa})]$$

where N_AA_, N_aa_ and N_Aa_ are the individual counts of genotypes AA, aa and Aa, respectively [8].

F is positive when there is ancestry-related assortative mating and F increases with the ancestry information of the marker. Previously [9], we considered the situation of an ancestrally admixed population and showed that the expected value of F, E(F) for a genetic marker is given by:

$$E\left(F\right)=\sigma^{2}\rho \delta^{2}/p*q*$$

where σ^2^ is the variance of genetic ancestry (in this case the proportion of ancestry from one of the progenitor populations), ρ is the correlation in ancestry between spouse-pairs, δ^2^ is the squared marker allele frequency difference between the two ancestral populations and p* and q* = 1-p* are the allele frequencies in the admixed population. This formula shows directly the relationship between F and the squared marker allele frequency difference, which in this case is the relevant measure of strength of association of the marker with ancestry. In fact, defining X as genetic ancestry of an individual and S as the presence of an allele at a marker locus, it can be shown that the correlation of X and S is given by:

$$Correl\left(X,S\right)=\sigma \delta /{\left(p*q*\right)}^{1/2}$$

and

$${\left[Correl\left(X,S\right)\right]}^{2}=\sigma^{2}\delta^{2}/p*q*$$

so that

$$E\left(F\right)=\rho {\left[Correl\left(X,S\right)\right]}^{2}$$ (1)

For the case we consider here, genetic ancestry is continuous and not assumed to represent admixture from ancestral populations. Rather, it is represented by principal components of the genetic marker data. However, there is a direct analogy based on formula (1) above. Specifically, the correlation of genetic ancestry X and SNP S is directly related to the loading of that SNP on the PC score. Hence, we expect the same linear relationship between F and the square of the PC loading for that SNP.

Therefore, to demonstrate the impact of ancestry-related assortative mating, we performed a linear regression analysis, using the F value for each SNP as the dependent variable and the square of the PC loading for that SNP as the independent variable. The F values for the 246,190 SNPs were calculated, and the regression of F on the square of the PC loading for each SNP was performed using a random sample of 100 unrelated participants from each generational cohort. These 100 unrelated participants were a subset of the 333 unrelated participants in the original and offspring cohorts used for deriving the PCs from the principal component analysis. The 100 unrelated participants in the third generation cohort were a random unrelated sample from that cohort.

We also previously showed [9] that under ancestry-related assortative mating, the expected value of the LD parameter, D, for two markers, is given by:

$$E\left(D\right)=\delta \varphi \sigma^{2}$$ (2)

where δ and φ are the ancestral allele frequency differences for the two markers. Again define X as an individual’s genetic ancestry, S as the presence of a particular allele at the first marker and T the presence of a particular allele at the second marker. From the derivation of formula (1) above we can write:

$$\delta =Correl\left(X,S\right){\left(p*q*\right)}^{1/2}/\sigma$$

and

$$\varphi =Correl\left(X,T\right){\left(r*s*\right)}^{1/2}/\sigma$$

where r* and s* = 1-r* are the allele frequencies at the second marker. Formula (2) can then be rewritten as:

$$E\left(D\right)=\left[Correl\left(X,S\right)\right]\left[Correl\left(X,T\right)\right]{\left(p*q*r*s*\right)}^{1/2}$$ (3)

As we described above, in the case where genetic ancestry X is represented by a principal component, Correl(X,S) reflects the PC loading for that marker; hence, we also expect a linear relationship between D and the product of the PC loadings for the pair of markers.

Therefore, to demonstrate the effect of assortative mating on LD, we performed a linear regression analysis with D as the dependent variable and the product of PC loadings as the independent variable. For computational efficiency in the regression analysis, a subset of 1000 SNPs was randomly selected, and D values were computed between SNPs on different chromosomes (N = 469,461) for the same individuals from each generation. We calculated D values between SNPs on different chromosomes, because these SNPs are unlinked. The D values were calculated and regressed on the product of the loading on PC1 for the first SNP and the loading on PC1 for the second SNP in the random sample of 100 unrelated participants from each generation. The same subset of participants used in the F regression described above was utilized for this analysis. This analysis was repeated regressing D values on the product of the loading on PC2 for the first SNP and the loading on PC2 for the second SNP.

### HWD and LD for SNPs related to height

Height is a highly heritable trait for which we also observed a significant spouse correlation. Large scale GWA studies of height have identified a substantial number of height quantitative trait locus (QTL) SNPs [42]. For comparison to our results for ancestry, we therefore decided to also examine evidence of HWD and LD for these height SNPs. Among 154 genome-wide significant SNPs reported in [42], 36 were genotyped on the Affymetrix arrays; an additional 118 were successfully imputed (r^2^>.80) from HapMap release 22. For the HWD and LD analyses, for the imputed SNPs we assigned the most likely genotype for each SNP/individual. For regression analyses of F and D, instead of PC-loadings for the independent variables, we used the SNP regression coefficients (betas) as reported in [42] as the measure of strength of relationship of the SNP to the trait (height). Specifically, in the analysis of F, we used the squares of the betas, and for the regression on D we used the product of the betas for the two SNPs. For the height SNPs that were genotyped and included in the PC analysis, we also had PC-loadings. However, for the height SNPs that were imputed, we did not. To create “quasi PC-loadings” for these imputed SNPs, we performed a regression analysis of SNP genotype on PC1 and used the regression coefficients. We did the same for the genotyped SNPs for consistency.

### Comparison of spouse kinship estimates with and without adjustment for genetic ancestry

We evaluated spouse-pair kinship estimates adjusting for genetic ancestry using PC-Relate software [33] and compared these estimates to those obtained without adjusting for genetic ancestry using GCTA software applying the default options with LD pruning at r-squared>0.3 and requiring minor allele frequency (MAF) > 0.05[31, 32]. Probability density functions for kinship estimates in the original and offspring cohorts were generated separately.

## Results

### Principal components analysis–characterizing European/West Asian ancestry

**Fig 3:**
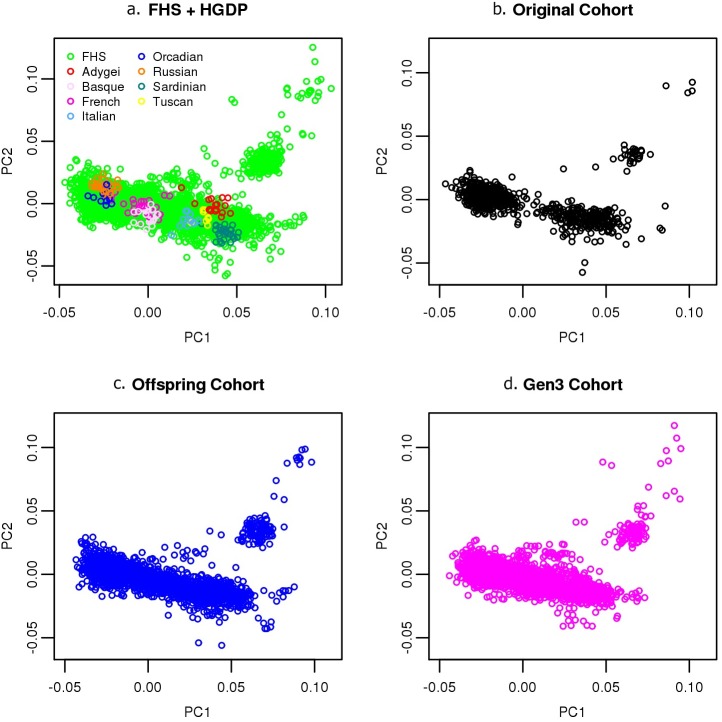
Principal components analyses on FHS participants. Principal components analysis on FHS participants with: Top left: Fig 3a, FHS and HGDP participants projected. Top right: Fig 3b, original cohort participants projected. Bottom left: Fig 3c, offspring cohort participants projected. Bottom right: Fig 3d, third generation cohort participants projected.

PC analysis based on 339 FHS participants was performed and the remaining FHS and the HGDP individuals were projected onto the first two principal components (Fig 3A). From the HGDP individuals, the first PC approximates the usual northwest-southeast cline in Europe, with Orcadians towards the left of the figure and Italians and Sardinians towards the right. The other prominent feature is a flow of points forming a discrete cluster along PC2 into the upper right corner of the diagram, which from other studies would indicate increasing Ashkenazi ancestry [43–45].

Separating the PC results by generation demonstrates a highly clustered distribution for the first generation (Fig 3B). In addition to the participants clustered along PC2 (Y-axis), there are two separated clusters along PC1 (X-axis). The two major clusters along PC1 likely reflect highly endogamous mating within the Northern/Western and Southern European subgroups. Based on historical demography and recent US Census data of Framingham, the Northern/Western European subgroup is likely comprised primarily of individuals of Irish, English, French and German ancestries, and the Southern European subgroup is likely comprised of primarily Italian ancestry [29, 46]. The two, almost discrete clusters seen along PC1 for the first generation (original cohort) start to merge into a single cluster in the second (offspring cohort) (Fig 3C) and more strongly in the third (Fig 3D) FHS generations, reflecting increased exogamy between Northern/Western and Southern Europeans over time. By contrast, the Y-axis clustering (PC2) appears to remain into the third generation. This suggests a slower rate of exogamy between the Ashkenazi and European subgroups.

### Characteristics of the FHS cohorts

In Table 1 we provide summary statistics for the various traits considered, including age, height, weight, BMI, SBP, and DBP. The age at enrollment of the offspring cohort was comparable to the original cohort because recruitment occurred several decades after enrollment of the original cohort. The offspring were slightly taller (P<0.05) and heavier (P<0.05) at entry into the cohort than their parents. The offspring had higher average BMI (P<0.05) and in particular the male offspring had higher BMI than their counterparts in the original cohort (P<0.05). SBP and DBP were both significantly lower in the offspring cohort (females and males). This may reflect the fact that 3.3% of the offspring cohort participants were on hypertension medication at the baseline exam, while none of the original cohort participants were treated at the baseline examination.

**Table 1:** Summary characteristics of the FHS cohorts’ spouse-pairs

	Original cohort (Gen1)		Offspring cohort (Gen2)	
Variable	Females (N = 124)	Males (N = 124)	Females (N = 755)	Males (N = 755)
Age in years at recruitment: mean (SD) *	34.85 (4.13)	36.49 (4.32)	35.75 (8.05)	37.90 (8.32)
Weight in pounds: mean (SD) *,**,***	131.29 (20.53)	169.39 (25.66)	136.94 (25.61)	181.97 (26.29)
Height in inches: mean (SD) *,**,***	62.86 (2.35)	68.59 (2.87)	63.73 (2.30)	69.13 (2.66)
BMI in kg/m^2^: mean (SD) *,***	23.35 (3.44)	25.28 (3.23)	23.67 (4.13)	26.75 (3.48)
Systolic Blood Pressure: mean (SD) *,**,***	122.10 (13.39)	129.18 (12.81)	116.78 (14.56)	125.78 (13.84)
Diastolic Blood Pressure: mean (SD) *,**	77.39 (9.39)	82.49 (8.81)	75.24 (9.76)	81.83 (9.55)

SD - standard deviation

* P< 0.05 comparing original cohort to the offspring cohort

** P<0.05 comparing females in the original cohort to females in the offspring cohort

*** P<0.05 comparing males in the original cohort to males in the offspring cohort

### Spouse correlations

Interclass correlations for spouse-pairs calculated separately for the first two generations (N = 124 and 755, respectively) were generated for age, the various anthropometric, and blood pressure measures along with the PC scores (Table 2). As expected, spouse correlations for age are high in both generations, but lower in the parent generation (r = 0.64, P<0.001) than the offspring generation (r = 0.91, P<0.001). Also as expected, there are moderate correlations for height between spouses, higher in the original cohort (r = 0.45 (P<0.001) than in the offspring cohort (r = 0.27, P<0.001). Correlations for weight are more modest, but still significant and again higher in the original cohort (r = 0.28, P = 0.002) than the offspring cohort (r = 0.16, P<0.001). Correlations for the two blood pressure measures were not different from 0 in the original cohort (r = -0.06, P = 0.54 for SBP and r = -0.03, P = 0.75 for DBP), but slightly positive and significant in the offspring cohort (r = 0.16, P<0.001 for SBP and r = 0.14, P<0.001 for DBP).

**Table 2:** Spouse Correlations in the FHS cohorts.

	Original cohort (Gen1)		Offspring cohort (Gen2)	
	Correlation (95% CI)	P-value	Correlation (95% CI)	P-value
Age	0.64 (0.52,0.73)	9.7x10^-16^	0.91 (0.90,0.93)	8.5x10^-298^
Weight	0.28 (0.11,0.43)	1.9x10^-3^	0.16 (0.09,0.23)	1.2x10^-5^
Height	0.45 (0.29,0.58)	1.9x10^-7^	0.27 (0.20,0.34)	8.4x10^-14^
BMI	0.17 (-0.01,0.34)	0.06	0.18 (0.11,0.24)	1.1x10^-6^
SBP	-0.06 (-0.23,0.12)	0.54	0.16 (0.09,0.23)	8.4x10^-6^
DBP	-0.03 (-0.20,0.15)	0.75	0.14 (0.07,0.21)	1.4x10^-4^
PC1	0.73 (0.63,0.80)	7.5x10^-22^	0.38 (0.32,0.44)	5.3x10^-28^
PC2	0.80 (0.72,0.85)	1.4x10^-28^	0.45 (0.39,0.51)	2.4x10^-39^

BMI–Body mass index

SBP–Systolic blood pressure

DBP–Diastolic blood pressure

PC1 –First principal component

PC2 –Second principal component

On the other hand, the spouse correlations for ancestry, as represented by PC1 and PC2, are strikingly positive and significant, especially in the first FHS generation, with values of r = 0.73 (P<10^−21^) and r = 0.80 (P<10^−27^) for PC1 and PC2, respectively. The pattern is similar in the offspring generation, although the correlations are attenuated compared to the prior generation, r = 0.38 (P = 10^−27^) for PC1 and r = 0.45 (P = 10^−38^) for PC2. In both generations of the FHS, the ancestry correlation of spouses far exceeds the correlations observed for the anthropometric and clinical traits, indicating that ancestry has been the most significant factor related to spouse choice apart from age. The reduced spouse ancestry correlations in the offspring generation reflect decreased endogamy over time.

**Fig 4:**
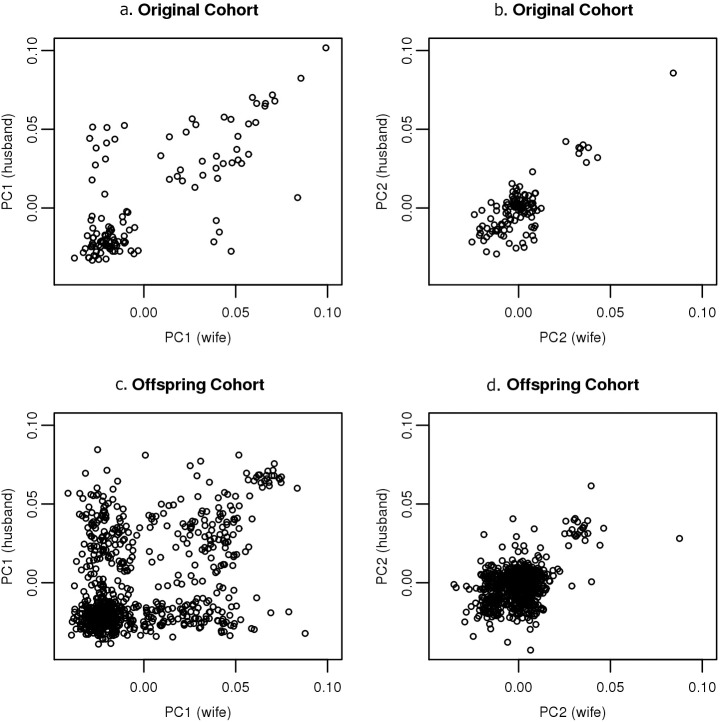
Scatter plots of spouses for PC1 and PC2, by generational cohort. **Scatter plots of spouse-pair male versus female:** Top left: Fig 4a, PC1 in the original cohort. Top right: Fig 4b, PC2 in the original cohort. Bottom left: Fig 4c, PC1 in the offspring cohort. Bottom right: Fig 4d, PC2 in the offspring cohort.

Fig 4 illustrates the ancestry-related assortative mating that has been present in Framingham over several generations. Each sub-figure is a scatter plot of PC scores for husbands versus wives for either PC1 or PC2, for the original cohort or the offspring cohort. The high correlation of PC scores between spouses as well as the clustering is quite apparent in the original cohort for both PC1 (Fig 4A) and PC2 (Fig 4B). In the original cohort, the clustering of spouses into Northwestern and Southern European subgroups is quite strong, with only a few spouse-pairings occurring between the two subgroups (with somewhat more unions between Southern European men and Northwestern European women than the opposite). The scatter diagram for PC2 in the original cohort also reveals strong endogamy, with few, if any, unions between clusters.

The second set of plots in Fig 4, characterizing the spouse-pairs of the offspring cohort, reveals similar patterns to the original cohort, but with less striking clustering, in particular for PC1. While again we see evidence of assortative mating based on ancestry, we also see a higher rate of exogamy compared to the original cohort, with a greater number of unions between Northwestern and Southern Europeans (Fig 4C). As in the top corresponding figure, we again see a higher rate of unions between Southern European men and Northwestern European women than the opposite (Fig 4C). Fig 4D, depicting PC2, once again illustrates a relatively higher rate of endogamy for the Ashkenazim.

### Impact of ancestry-related assortative mating on Hardy-Weinberg equilibrium and linkage disequilibrium

As expected, the ancestry-related positive assortative mating resulted in both within-locus (HWD due to homozygote excess) and between-locus (LD) observable effects. We examined HW deviations in each of the three generations of the FHS by calculating the standardized homozygote excess measured by F for each SNP, and performed regression analysis with F as the dependent variable and squared PC loading as the independent variable (Table 3). In all three generations, the regressions are significant, both for PC1 and PC2. As expected, the magnitude of the regression coefficient and strength of the association is greatest for the participants in the original cohort and decreases continuously in the second (offspring cohort) and third generations (Table 3), although the decrease is much more dramatic between the last two generations. This result is consistent with the reduction of endogamous mating over the past 60 years in Framingham as we observed in the spouse PC correlations in the first two (original and offspring) FHS cohorts. HW deviations reflect assortative mating patterns from the previous generation. Hence, the high level of HW deviation observed in the original cohort suggests that spouse ancestry correlations were even stronger in the generation that gave rise to the original cohort (parents of the original cohort participants), although a more rapid decline in endogamy is inferred to have occurred between the original and offspring generations than in earlier generations.

**Table 3:** Regression of Hardy–Weinberg disequilibrium parameter F on squared SNP PC Loadings.

Principal Component	Generation	Estimate ± s.e. (x 10^−3^)	T-value	P-value
PC1	Gen1	4.148±.142	29.24	1.2x10^-187^
	Gen2	3.700±.141	26.24	2.8x10^-151^
	Gen3	0.968±.141	6.89	6.1x10^-12^
PC2	Gen1	2.689±.139	19.28	9.3x10^-83^
	Gen2	2.106±.139	15.18	5.0x10^-52^
	Gen3	0.462±.138	3.34	8.4x10^-4^

Gen1—Original cohort

Gen 2—Offspring cohort

Gen3—Third generation cohort

PC1 –First principal component

PC2 –Second principal component

s.e.–standard error

Similarly, we examined the LD parameter D for pairs of unlinked SNPs as a function of the product of PC SNP loadings via regression analysis (Table 4). Again we see a pattern very consistent with those in Table 3 for HW deviations. With the exception of PC2 in generation 3, the regressions are significant across each of the 3 generations of the FHS, with attenuation of the regression coefficients in each succeeding generation, particularly between the second and third generations. As opposed to HW deviations, which depend only on mating patterns of the prior generation, LD can persist over many generations, depending on the linkage relationship between markers, even under random mating. Nonetheless, we did see a reduction of the regression of the LD parameter D on the product of the PC loadings across generations for both PC1 and PC2, again consistent with the relaxation of endogamy over the same time period.

**Table 4:** Regression analysis of linkage disequilibrium parameter, D on the product of the PC loadings for unlinked SNPs.

Principal Component	Generation	Estimate ± s.e. (x 10^−3^)	T-value	P-value
PC1	Gen1	1.195±.0241	49.65	<1x10^-324^
	Gen2	0.985±.0241	40.89	<1x10^-324^
	Gen3	0.183±.0242	7.56	4.1x10^-14^
PC2	Gen1	0.734±.0243	30.17	2.9x10^-200^
	Gen2	0.559±.0243	22.96	5.9x10^-117^
	Gen3	0.022±.0244	0.91	0.36

Gen1—Original cohort

Gen 2—Offspring cohort

Gen3—Third generation cohort

PC1 –First principal component

PC2 –Second principal component

s.e.–standard error

### Impact of assortative mating for height

Height is a trait with high heritability and also showed significant spouse correlations in our sample (Table 2: r = 0.45 and r = 0.27 in original and offspring generations, respectively). Direct assessment of HWD and LD for the 154 height SNPs was complicated by the fact that height is also correlated with genetic ancestry (PC1). Specifically, we calculated a weighted genetic risk score (GRS) from the 154 SNPs based on the betas (regression coefficients) as reported in [42]. Measured height is positively correlated with PC1 (r = 0.21 and 0.11 in the original and offspring cohorts, respectively); the height GRS shows even higher correlations with PC1 (r = 0.29 and 0.25 in the original and offspring cohorts, respectively). This means that at least some of the height SNPs could show HWD and/or LD due to their correlation with ancestry (PC1). Therefore, we constructed a regression model with F as the dependent variable and both height beta-squared and quasi PC-loading squared included as independent variables. Similarly, we included products of both betas for each pair of SNPs and products of quasi PC1-loading for each SNP pair as independent variables in the regression model for D.

For the regression model on F, neither the beta-squared nor quasi PC1-loading-squared terms are statistically significant. This is not surprising given the limited number of SNPs for this analysis and the fact that assortative mating has less impact on HWD than on LD. By contrast, there is a highly significant effect of the quasi PC1-loading products on D (t = 11.63, P<2.2x10^-16^; t = 5.21, P = 1.9x10^-7^; t = 4.23, P = 2.3x10^-5^ for original, offspring and third generation cohorts, respectively); however there is no significant effect of the beta products (t = 0.091, P = 0.93; t = -0.019,P = 0.98; t = 0.058, P = 0.95 for the three cohorts, respectively). Thus, the impact of assortative mating for height on population structure (i.e. LD) appears to be dramatically less than assortative mating for ancestry.

### Comparison of spouse kinship estimates with and without adjustment for genetic ancestry

We first compared the spouse kinship coefficients estimated using GCTA (without ancestry adjustment) to those using PC-Relate (with ancestry adjustment). We categorized individuals into three groups based on scores for PC1 and PC2 –those representing an Ashkenazi cluster, those representing a Northwestern European cluster, and those representing a Southern European cluster. It is important to note that there are individuals who are mixed between these groups, so to some degree the clustering is imprecise; however it is still useful to demonstrate the impact of ancestry adjustment.

**Fig 5:**
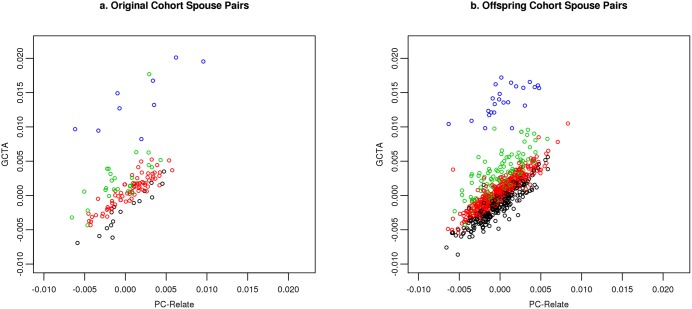
Comparison of kinship estimates without ancestry adjustment (using GCTA) versus with ancestry adjustment (using PC-Relate). (a) Original cohort. Blue color represents Ashkenazi spouse pairs; red color represents Northwestern European spouse pairs; green color represents Southern European spouse pairs; black color represents spouse pairs of different ancestry. (b) Offspring cohort. Blue color represents Ashkenazi spouse pairs; red color represents Northwestern European spouse pairs; green color represents Southern European spouse pairs; black color represents spouse pairs of different ancestry.

A clear pattern of difference between ancestry adjusted and ancestry unadjusted kinship estimates is observed (Fig 5), both in the FHS original cohort and offspring cohort. While the mean kinship varies little among the various ancestry groupings, the mean kinship is clearly increased for the ancestry endogamous pairs–with the highest mean for the Ashkenazi pairs, followed by the Southern European pairs; the Northwestern European pairs appear to have the smallest increase, likely because the majority of the sample is of Northwestern European ancestry. The figure clearly shows the upward bias in estimation of kinship when ancestry is not taken into account.

**Fig 6:**
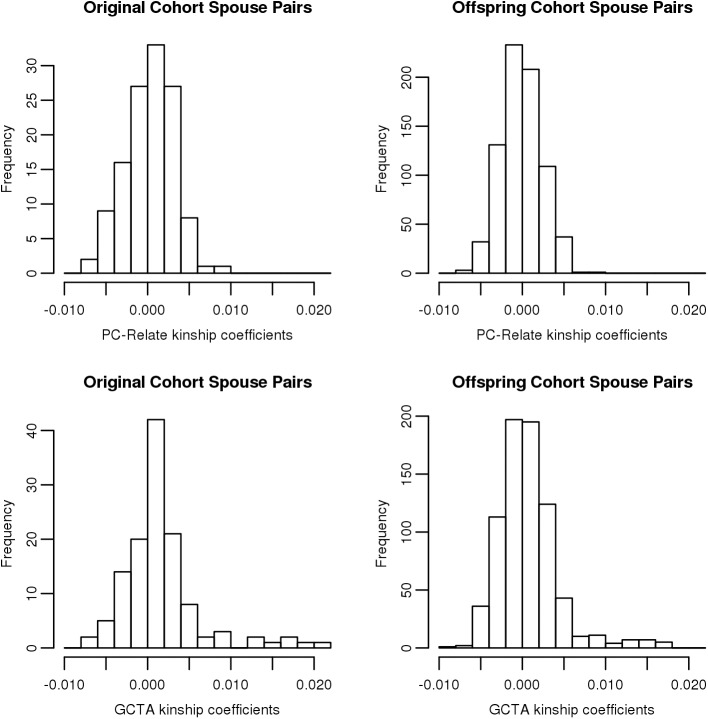
Probability density functions of spouse kinship estimates comparing results from GCTA (unadjusted for genetic ancestry) to those from PC-Relate (adjusted for genetic ancestry) in the FHS original cohort and offspring cohort.

These results are also reflected in histograms of kinship coefficients for the original and offspring cohorts (Fig 6). The unadjusted kinship estimates show a large right tail, likely due to the endogamy seen in the Ashkenazim in both the original and offspring cohorts, as observed in Fig 5. In both cohorts, mean kinship estimates as well their standard deviations (SD) are higher when not adjusting for genetic ancestry (original cohort: mean = 0.001653, SD = 0 .00470; offspring cohort: mean = 0.00070, SD = 0.00367) than when adjusting for genetic ancestry (original cohort: mean = 0.00033, SD = 0.00286; offspring cohort: mean = -0.0001, SD = 0.00241). For the original and offspring cohorts, the ancestry unadjusted means are significantly greater than 0 (P<10^−4^ and P<10^−7^, respectively), while the ancestry adjusted means are not significantly greater than 0.

## Discussion

We have conducted the first multi-generational analysis of mating patterns for a U.S. population, based on FHS participants from Framingham, Massachusetts. Our findings have significance for a variety of reasons: they reflect demographic patterns that have occurred over the past 60 years and reveal changes in those patterns over time; document the genetic implications of mating patterns and changes in mating patterns over time; and provide a warning about potentially simplistic assumptions in the genetic modeling of human populations.

Soon after World War II, when the FHS was initiated, the demography of Framingham was largely white and middle class, with recent growth from the baby boom. It largely reflects the ancestral make-up of Massachusetts, which is both Northern and Southern European. According to the U.S. Census Bureau 2010 American Community Survey, among whites, the predominant ancestries in Framingham are: Irish (23%), English (13%), French (10%), other Northern European (27%), Italian (18%), and other Southern European/Mediterranean (8%). Similarly, in the FHS, the ancestries are predominantly Irish (15%) or other British (20%), Italian (19%), and other Western European (32%) [29]. There is also a sizeable Ashkenazi population in Framingham constituting perhaps 5% of the total population [35].

Mate choice reflects a large number of factors including local geodemographics, social class, nationality, ethnicity, religion, anthropometric traits such as height and weight, as well as behavioral characteristics. Our findings clearly document the strong endogamy that existed in Framingham prior to World War II. These patterns may also have reflected neighborhood characteristics, and the tendency for unions to occur locally. Intermixing between participants with Northwestern and Southern European ancestries was relatively uncommon in the original cohort, but increased in subsequent generations. Similarly, there was a strong tendency towards endogamy in the participants that clustered along PC2, implicitly those with Ashkenazi ancestry.

The strong endogamy was also clearly evident in the large deviations from HW of SNPs correlated with either of the first two PCs, as well as the dramatic evidence of LD between unlinked markers correlated with those same PCs. It is the strength of the regressions of F and D on these PC loadings that demonstrates directly the impact of the ancestry-related assortative mating, as other factors creating HW deviations and LD (e.g. inbreeding and genotyping error) would not produce such a pattern [10].

The spouse genetic ancestry correlations observed in the original cohort were remarkably high, even greater than spouse correlation in age. This assortment was also evident in the strong HW deviations and LD observed in the subsequent generation (offspring cohort). In fact, the regression analyses on HW and LD parameters revealed evidence of even stronger spouse ancestry correlations in the generation that gave rise to the original Framingham cohort.

We also observed a consistent decay in endogamy in Framingham over 6 decades, reflected both in the spouse correlations for genetic ancestry and in the HW and LD deviations. While strong clustering was observed in the original FHS cohort, one can see a clear decay in such clustering in subsequent generations, and also an increase in the number of unions between the clusters in the offspring cohort compared to the original cohort.

Although examination of genetic ancestry correlation in spouse-pairs provides direct evidence of assortative mating, indirect evidence can also be obtained by examination of HW and LD patterns of SNPs as a function of their correlation with genetic ancestry, as we have also demonstrated here and previously [9, 10]. Indeed, for large population cohorts where spouses have not been identified, one can examine both the evidence of population structure by PC analysis and correlation of HW disequilibrium and LD statistics with ancestry-informative SNPs as a function of age. As we have seen in the Framingham population, genetic structure can vary significantly by age as a consequence of changes in social demography and mating patterns. Also, we have only analyzed LD patterns for pairs of SNPs located on different chromosomes. Because LD persists over time, especially when SNPs are linked, we anticipate considerable residual LD for many nearby linked markers as a consequence of strong assortative mating in the past.

Although we have provided clear evidence of HW deviations and LD in the FHS, it is still important to note that the strength of such deviations depends on the degree of allelic differentiation (for example, as measured by Wright’s fixation index F_ST_) among the ancestral groups contributing to the current population. Because the large majority of white ethnic groups have only modest F_ST_ values on average, it is expected that most SNPs typed in a sample similar to the FHS would not demonstrate significant homozygote excess, especially in a sample that is not large.

However, for some SNPs, i.e. those with greater European differentiation, the spouse genotype correlation and HW deviation can be substantial. Genome-wide association studies, while often correcting for the inclusion of close relatives, generally do not correct for inclusion of spouses. Close relatives may be correlated for genotypes across the entire genome. By contrast, the genotype correlation between spouses may be proportional to how correlated a marker is with genetic ancestry, and therefore marker specific. So, for ancestry-related SNPs, ignoring the genotype dependence between spouses could also lead to inflated statistical significance, as it does when closely related individuals are included.

The power of the ancestry-related assortative mating analysis presented here is due to the strong relationship of some SNPs with genetic ancestry in Europeans (i.e. those with the strongest impact on the PC scores). By contrast, a similar analysis of SNPs underlying a phenotypic trait undergoing assortative mating would not typically show such evidence of HWD and LD because the individual SNP effects on the trait are too modest. For example, we also examined height, a highly heritable trait with a significant spouse correlation, in the same fashion, but found no evidence of correlation of the LD measure D among the pairs of 154 height-associated SNPs with the product of their regression coefficients on height, but did find a significant correlation of LD for the same pairs of SNPs with their PC1-loading products.

On the other hand, genotyping error can also have a strong influence on HWD (but not LD). For example, in our original SNP QC analysis, we examined the proportion of SNPs with a significant HW test (P < .0001) as a function of call rate. For SNPs with call rate of at least 99%, 0.2% had a significant HW test. For SNPs with call rate of 98–99%, 1.2% had a significant HW test. For SNPs with call rate <97%, 5.9% had a significant HW test. However, the HWD of these SNPs is unlikely to be correlated with PC1 or PC2.

We also demonstrated that ancestry adjustment can influence kinship estimates, particularly those for pairs of individuals less than close (e.g. third degree) relatives. Because modern heritability estimation approaches depend on kinship estimates in this range, caution needs to be exerted in interpreting those heritability estimates if ancestry is not considered. In other words, heritability estimates obtained using kinship estimates without ancestry adjustment may be biased, depending on the degree to which genetic ancestry is related to the trait being examined.

A remaining and important question is the degree to which our observations in Framingham generalize to other populations, both within the U.S. and elsewhere. The issue becomes an even greater concern as populations from different geographic locations are combined in meta-analyses. The 2010 US census collected ancestry information, and maps created from that information reveal patterns of national diversity as well as changes over time due to migrations. While unions historically have been preferentially local, increased movements of the population over past decades are contributing to the decay of local endogamy, as seen in Framingham. In addition to geography, which itself is correlated with ancestry, other social factors may have influenced mate selection such as race/ethnicity, nationality, education, socioeconomic status and religion. While the exact patterns we have seen in the FHS may not replicate elsewhere, it is likely that similarly complex mating patterns, including spouse correlations related to genetic ancestry, are a common feature, and need to be addressed in any population study.
